# A study on the influence of product environmental information transparency on online consumers’ purchasing behavior of green agricultural products

**DOI:** 10.3389/fpsyg.2023.1168214

**Published:** 2023-04-11

**Authors:** Shaoling Fu, Ruili Ma, Guangyao He, Zhiyi Chen, Hua Liu

**Affiliations:** ^1^College of Economics & Management, South China Agricultural University, Guangzhou, China; ^2^Chongqing Huadi Zihuan Technology Co., Chongqing, China

**Keywords:** product information transparency, competence trust, benevolence trust, online purchasing behavior, green agricultural products

## Abstract

**Introduction:**

In 2020, the outbreak of COVID-19 has forced consumers to shift their consumption patterns online. However, the problem of online fraud in green agricultural products seriously undermines consumer trust and is detrimental to the sustainable consumption of green agricultural products. Therefore, it is particularly important to enhance consumers’ trust in online sellers. This study aims to investigate how the product environmental information transparency(soil information transparency and water information transparency) affects online consumers’ purchasing behavior of green agricultural products.

**Methods:**

This study constructs a theoretical framework of “product environmental information transparency - online consumer trust - online purchase behavior”.We conducted an online randomized questionnaire to collect data from a sample of 512 consumers who had experience buying green agricultural products online fitted a structural equation model (SEM).

**Results:**

The results show that (1) the two dimensions of product environmental information transparency have different effects on different dimensions of online consumer trust. Among them, soil information transparency has a significant positive effect on competence trust, while it does not have a significant positive effect on benevolence trust. Water information transparency has a significant positive effect on both dimensions of online consumer trust, (2) online consumer trust has a significant positive effect on online consumer purchase behavior, and (3) competence trust has a significant positive effect on benevolence trust.

**Discussion:**

Our study shows that consumer trust in merchants is significantly enhanced by increasing the transparency of environmental information about green agricultural products. different dimensions of environmental information transparency have different effects on different dimensions of online consumer trust. Product information transparency is proposed as a tool for producers to use in the online marketing of green agricultural products. Consumers’ access to information can be improved through online public disclosure of environmental quality indicators in the production process of green agricultural products, and ultimately enhance online consumption of green agricultural products.

## 1. Introduction

Environmental pollution is a major obstacle to achieving sustainable development and has received widespread attention (Wang et al., 2021; Yang et al., 2022). Existing studies indicate that emissions from agricultural production are inextricably linked to pollution and climate change (Rehman et al., 2021). GHG emissions from food distribution processes are also particularly important for the overall agricultural environment, especially in short transport supply chains where electric vehicles can reduce GHG emissions and consumers are willing to pay premium prices for products provided in a more sustainable manner (Galati et al., 2022, 2023). China’s food system is the largest contributor to GHG emissions, accounting for about 13.5% of the total global food system. In developing countries, China’s food system emissions increased by 41% from 1990 to 2015, much higher than other countries (Crippa et al., 2021). In addition, China’s agricultural soil contamination rate exceeded 19% in 2014 alone, mainly in the form of heavy metal-contaminated agricultural soils.^1^ The Chinese government states that it should vigorously develop green agricultural products and follow an environmentally friendly and sustainable development path. It also proposes to achieve the goal of carbon neutrality by 2060. Therefore, promoting the sustainable development of green agricultural products plays an important role in reducing environmental pollution and achieving the goal of carbon neutrality.

To achieve sustainable development in the field of agriculture and food business, each stakeholder in the chain plays an important role, and consumers, as an important link, play a key role in participating in these processes (Galati and Adamashvili, 2023). Different national populations are self-aware and are influenced by factors such as sustainability of food production processes, food composition, and labeling in their food consumption purchase decisions (Salvatore et al., 2022). At present, the development of green agricultural products market is still facing many problems, which seriously hinders its sustainable development. Firstly, the price of green agricultural products is high and it is difficult to screen them. Second, consumers’ awareness of green agricultural products is insufficient. In the actual purchase scenario, the limited label space cannot provide sufficient information, making consumers’ original ambiguous perceptions not only unable to be corrected in the purchase scenario, but may be further amplified, which in turn weakens their purchase motivation (Rana and Paul, 2017). Third, consumers lack sufficient trust in the authenticity of green produce labels and whether the production process is strictly implemented according to green production practices (Macready et al., 2020). The key to overcome these obstacles lies in how to establish an effective signaling mechanism to reduce information asymmetry between producers and consumers.

With the development of “Internet+” e-commerce platforms and the normalization of COVID-19 prevention and control, online platform has gradually become one of the important channels for the sales of green agricultural products. However, with the gradual expansion of online consumption, consumer satisfaction has also encountered a crisis. Alaimo et al. (2022) measured consumer satisfaction with online food purchases during COVID-19 and concluded that consumer satisfaction in using online purchases was influenced by the surrounding environment and situational factors, and although consumers had a good experience with different stages of the online purchase process, their expectations of the final outcome were often not met. Expectations of the end result were often not met. In 2021, the Q3 e-commerce user complaints monitoring report showed that online fraud (9.85%), product quality (8.77%), after-sales service (6.21%), online fake sales (5.49%), overlord clauses (5.34%), delivery problems (4.87%), false promotions (4.31%) and so on have become hot complaints of online consumption at this stage (https://www.100ec.cn/detail--6602109.html). Incidents of online fraud and information asymmetry between buyers and sellers can reduce consumer trust in product labels and attributes. Failure to properly manage consumer trust will result in the economic “Gresham’s phenomenon,” i.e., “the bad currency drives out the good currency” (Synnes Emblemsvåg and Emblemsvåg, 2022), reducing the market share of green agricultural products with higher real value. This has seriously dampened the motivation of green agricultural producers and hindered the sustainable development of the online consumer market for green agricultural products. Therefore, how to reduce information asymmetry and enhance consumer trust is the key to achieve sustainable development of the market for green agricultural products.

Transparency of product information can reduce information asymmetry and thus enhance consumer trust in sellers (Fu et al., 2022). Compared with ordinary agricultural products, green agricultural products may have more stringent environmental requirements during the growing process, but this type of information is not within the scope of mandatory information disclosure by firms. Previous studies have also paid less attention to such non-mandatory disclosure information, i.e., the role of invisible information, and have mainly focused on the impact of visible information such as product prices (Katt and Meixner, 2020; Melovic et al., 2020; Wang et al., 2020; Zheng et al., 2021), packaging (Chang et al., 2021; Liang et al., 2022) and labels (Spognardi et al., 2021; Ma et al., 2022). Sustainable product labeling can have a significant impact on consumers’ purchasing decisions by providing enough information to obtain timely and reliable information about the environment in which food is grown (Giacomarra et al., 2021). Khan et al. (2021) suggested that signaling theory can contribute significantly to understanding the various antecedents and precursors that drive consumers’ green consumption behavior. Therefore, this study is based on signaling theory to investigate the impact of invisible information, namely the transparency of environmental information of green agricultural products, on online consumer trust and the impact of consumer trust on purchase behavior. According to Fu et al. (2022), online consumer trust dimensions were selected: competence trust and benevolence trust. To investigate the role of different dimensions of product environmental information transparency on online consumer trust, and the role of competence trust and benevolence trust on online consumer purchase behavior of green agricultural products, To provide a possible reference for the sustainable development of green agricultural products.

This paper aims to address the following three questions: (1) What is the effect of online environmental information transparency of green agricultural products on consumers’ competence trust and benevolence trust?, (2) What is the effect of online consumers’ competence trust and benevolence trust on consumers’ purchase behavior?, and (3) What is the relationship between competence trust and benevolence trust?

## 2. Theoretical analysis and research hypotheses

### 2.1. The relationship between soil information transparency and online consumer trust

In the context of B2C e-commerce, trust is very important for the sustainable development of online commerce. Trust is defined as “confidence in the reliability and integrity of an exchange partner” (Morgan and Hunt, 1994, p. 23). Considering the characteristics of green agricultural products, we refer to the existing research to classify trust into competence trust and benevolence trust (Chen, 2010). Competence trust refers to consumers’ trust that the online seller can provide high quality products or services, and consumers’ satisfaction with the competence, knowledge and skills of the online seller. On the other hand, benevolence trust refers to consumers’ belief that online sellers pursue their own interests as well as those of the consumer, which influences online consumers’ perceived trust (Han and Yan, 2019).

According to signaling theory, product information can be considered as a signal sent by online sellers to consumers, which influences consumers’ perceived trust and purchase behavior (Fu et al., 2022). In green product sales, Mezger et al. (2020) argued that there is an information asymmetry in online shopping, such as users cannot find out product characteristics and consumers are uncertain whether the actual quality of green products is reliable. The high level of uncertainty reduces online consumers’ trust in the seller. Therefore, sellers need to enhance trust by providing relevant product information that consumers need, which in turn leads to consumers’ willingness to purchase green products (Chen, 2007).

The product information that consumers are interested in can be divided into five specific categories: product origin, pesticide use, production characteristics, production environment and price information (Moser et al., 2012). Among these, environmental issues have a significant impact on consumers’ organic food purchasing behavior, among which soil contamination has a significant and positive impact on individuals’ daily organic food consumption (Laureti and Benedetti, 2018). Therefore, if online sellers can openly share information about the soil during the growth of organic produce to help consumers judge the quality of organic produce, consumers will believe that online sellers are capable of producing quality organic produce, which will increase consumers’ trust in the capabilities of online sellers. As a result, this study proposes the following hypothesis:

*H1a*: Soil information transparency has a significant positive impact on online consumer competence trust.

Benevolence trust is defined by Mayer et al. (1995) as “the extent to which a trustee is perceived to want to do good to the principal, apart from self-interest motives.” This means that the seller takes into account not only their own interests but also those of their stakeholders, such as the intention to be ecologically beneficial to consumers and the environment. At the same time, rapid land use change, especially soil erosion, is an important cause of the decline in the value of social and ecological services in China (Qi et al., 2014; Wang et al., 2022). Sellers can motivate their own behaviors and better promote their development as well as adjust their related actions in clarifying consumers’ attitudes, awareness levels, and decision-making processes (Salvatore et al., 2022), so disclosing information on soil-related indicators during the growth of green agricultural products is important. It helps to demonstrate that the online seller is genuinely concerned about the consumer and meets the consumers’ interests, thus enhancing the consumers’ perception of the sellers’ benevolence. Therefore, the following hypothesis is formulated in this study:

*H1b*: Soil information transparency has a significant positive impact on online consumer benevolence trust.

### 2.2. The relationship between water information transparency and online consumer trust

This study concludes that the greater the transparency of information on water sources, the greater the consumer’s trust in the online seller’s ability to deliver. Using rice as an example, research suggests that producers can contribute to food security by growing rice with more efficient irrigation techniques and higher quality water (Mullick and Das, 2021). Online sellers who disclose information about these water sources will encourage consumers to believe that the seller is able to provide quality green agricultural products, the ability to achieve environmental protection, which in turn increases consumers’ trust in the ability of online sellers. Therefore, the following hypothesis is formulated in this study:

*H2a*: Water information transparency has a significant positive impact on online consumer competence trust.

Water for production is one of the factors that affects the quality and safety of green agricultural products. When companies disclose indicators related to the water sources used in the production of green agricultural products, it helps to demonstrate that the online seller cares about the health of the consumer and has met the quality standards of the water sources used in the production of green produce, thus increasing consumer trust in the benevolence of the online seller. Han and Yan (2019) argued that the transparency of information can directly influence people’s risk perception, and thus consumer trust. Castro-González et al. (2021) empirically investigated the influence of environmental labeling information and product sustainability information on the existence of consumer trust in a selection of Spanish food products, and showed that product environmental factors have a positive influence on consumer trust. Therefore, the following hypothesis is formulated in this study:

*H2b*: Water information transparency has a significant positive impact on online consumer benevolence trust.

### 2.3. The relationship between online consumer trust and purchase behavior

This study concludes that consumers’ competence trust has a positive effect on online purchasing behavior for green agricultural products. In online shopping platforms, consumers are often unable to visualize products and therefore are concerned about potential quality issues with products. Competence trust is a prerequisite for consumers to judge sellers and make purchase decisions (Fu et al., 2022). Supply chain transparency can stimulate indirect reciprocity among consumers by increasing competence trust, benevolence trust, and integrity trust through improved perceived information quality (He, 2023). In online shopping situations, when consumers’ trust in a seller’s competence is established, they tend to trust the seller’s ability to provide safe, quality-compliant products and reliable product information, and these trusts can lead to online purchase behavior. Research shows that consumer trust in sellers’ competence has a positive effect on consumer purchase behavior in online shopping (Fu et al., 2022). Therefore, the following hypothesis is formulated in this study:

*H3a*: Online consumer competence trust has a significant positive impact on online purchase behavior.

This study concludes that consumers’ benevolence trust in online sellers has a significant positive effect on consumers’ online purchase behavior of green agricultural products. Since online shopping cannot be touched physically and consumers are faced with a multitude of consumer choices, benevolence trust facilitates a positive emotional connection with the seller and prevents consumers from turning to competitors’ products, thereby stimulating consumer purchase behavior. Mohan et al. (2021) investigated how online consumer trust affects consumer purchase intention and found that benevolence trust has a significant positive effect on consumer purchase intention. In contrast, Fu et al. (2022) found that online consumers’ benevolence trust in merchants can significantly influence purchase behavior. Therefore, the following hypothesis is formulated in this study:

*H4*: Online consumer benevolence trust has a significant positive impact on online purchase behavior.

### 2.4. Competence trust and benevolence trust

The detailed variable measurement items and sources are shown in Table 1. When consumers have a high level of trust in the ability of producers to deliver green agricultural products that meet quality requirements, they are more likely to believe that producers share their values, such as protecting the environment and being responsible to others. Consumer trust is a key prerequisite for establishing a market for green products, and there is a positive correlation between green product attributes (such as perception and perceived attributes) and consumer trust in purchasing green products (Khan et al., 2022). Based on this concept, consumers trust that the seller will protect the consumers’ basic rights and not compromise them for their own benefit. Fu et al. (2022) confirmed that competence trust promotes benevolence trust. Therefore, the following hypothesis is formulated in this study:

*H3b*: Online consumer competence trust has a significant positive effect on online consumer benevolence trust.

**Table 1:** Variable measure items and sources.

Construct	Items	Source
Soil information transparency	The online seller publicly provides information about the quality of the soil used in the production of green agricultural products	Liu (2013), Fu et al. (2022)
	The seller provides generally consistent information about the quality of the soil used in the production of green agricultural products on various channels	
	The online seller publicly shares consumer comments about the quality of soil in the production of green agricultural products	
Water information transparency	The seller compares the quality of the irrigation water used in the production of green agricultural products with the green quality standard online to illustrate its advantages and disadvantages	
	The online seller regularly updates information on the quality of irrigation water used in the production of green agricultural products	
	The online seller provides timely information about the quality of irrigation water used in the production of green agricultural products	
Competence trust	I believe that the online seller is a reputable business	McKnight et al. (2002), Xu et al. (2016), Mohan et al. (2021)
	I believe that the online seller knows the market in which they operate	
	I believe that the online seller’s green agricultural products are of good quality	
Benevolence trust	The online seller has my best interests in mind	
	The online seller wants to understand my needs and preferences	
	The online seller is genuinely interested in their customers	
Online purchasing behavior	I would recommend others to buy the online seller’s green agricultural products	Kang and Hustvedt (2014), Suki and Suki (2019), Talwar et al. (2021)
	I will increase the frequency and proportion of my purchases of green agricultural products from this online seller in the future	
	I still buy green agricultural products from this online seller regularly, despite having many choices	

**Figure 1:**
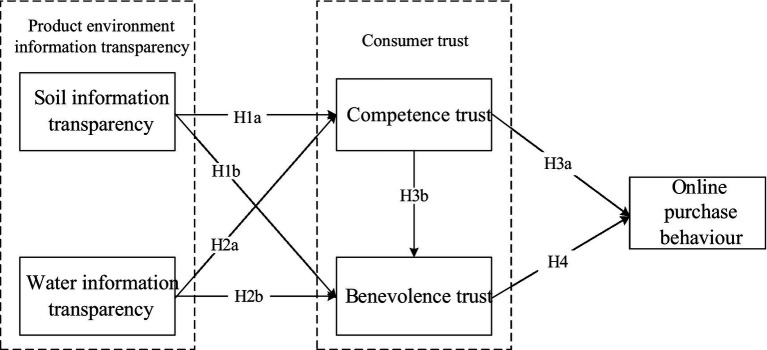
Theoretical model.

In summary, the theoretical model in this paper is shown in Figure 1.

## 3. Research methodology

### 3.1. Questionnaire design and data collection

In this paper, the questionnaire method was chosen for data collection. A 7-point Likert scale (1–7 for “strongly disagree” – “strongly agree”) was used to survey consumers with experience of buying green agricultural products online. Given the special circumstances of COVID-19, the official questionnaire was distributed through two online methods: (1) Questionnaires were distributed online through the social networking platform “WeChat,” mainly in the form of posting to WeChat friends circle and forwarding to WeChat groups and (2) Questionnaire Star, a questionnaire collection platform, was commissioned to randomly distribute questionnaires on shopping websites. A total of 570 questionnaires were collected, and after eliminating invalid questionnaires such as those with a response repetition rate of over 90% and those with too short a response time, the final number of valid questionnaires was 512, with a valid response rate of 89.8%.

### 3.2. Variable measurements

The questionnaire is adapted from published and well-established scales. The measurement of soil information transparency and water information transparency in this study is based on the scales proposed by Liu (2013) and Fu et al. (2022), which contain three measurement questions, respectively. Existing scales of online consumer trust are well established, and we divide online consumer trust into competence trust and benevolence trust, each containing three measure items, based on scales by Mcknight et al. (2002) and Xu et al. (2016), and more recently Mohan et al. (2021). To measure online consumer purchase behavior, we refer mainly to the content of the research scale by Kang and Hustvedt (2014); Suki and Suki (2019); Talwar et al. (2021), with three measure items (see Table 1).

### 3.3. Description of sample statistics

The basic characteristics of the sample are shown in Table 2, The survey targeted at consumers who had experience with purchasing green agricultural products online. The percentage of females in the sample is 63.7%, while the percentage of males is 36.3%. This may be related to the different roles played by men and women in family life, with women usually tending to be more concerned with dietary life and more inclined to make online purchases than men (Qing, 2017). This structure is reasonable. In terms of age, people aged 25 to 34 account for the largest proportion of the sample (48%), people aged 18 to 24 account for 26.9% of the sample, and people aged 35 to 44 account for 20.2% of the sample, in line with the structural scale characteristics of China’s Internet users.^2^ 70.9% of respondents hold an undergraduate degree. The sample covers a wide range of residents’ monthly income levels, with the largest group being below RMB 3,000 (22%), followed by RMB 5,001–8,000 (20.2%). The sample is reasonably representative.

**Table 2:** Basic characteristics of respondents.

Attributes	Options	Frequency	Percentage (%)
Gender	Female	313	63.7
	Male	199	36.3
Age	18–24	112	26.9
	25–34	249	48
	35–44	98	20.2
	45–54	36	4.5
	55–64	16	0.4
	≥65	1	0
Education	Junior middle school or below	5	0.4
	Senior middle school/polytechnic school/technical school	40	6.3
	Junior college	60	5.8
	Undergraduate course	349	70.9
	Master’s	51	13.9
	Doctorate	7	2.7
Monthly income (CNY)	Below 3,001	91	22
	3,001–5,000	98	14.8
	5,001–8,000	114	20.2
	8,001–10,000	78	13.5
	10,001–15,000	78	17
	15,001–20,000	34	8.1
	Over 20,000	19	4.4

In view of the possible problem of common method bias, Harman’s one-way test was used first, and the results of the unrotated exploratory factor analysis extracted a total of 10 factors with characteristic roots greater than 1, and the variance explained by the first factor is 32.67%, which is below the critical criterion of 50%, indicating that there is no serious problem of common method bias.

### 3.4. Reliability and validity analyses

SPSS 26.0 and Amos 24.0 software were used to test the reliability and validity of the scales. In Table 3, the Cronbach’s α values for all variables are above 0.70 and the CR values are above 0.70, indicating that the reliability of the scales used in this paper is high. The factor loadings and AVE values for each variable are above 0.50, indicating that the scale used in this paper has good convergent validity. Table 4 shows that the AVE square root of each construct (diagonal line) are higher than the corresponding correlation coefficients (values in the non diagonal line), which means that the scale has good discriminant validity.

**Table 3:** Analysis of sample reliability and convergent validity.

Construct	Items	Unstd	S.E.	Z-value	*P*	Std.	SMC	CR	AVE	Cronbach’s α
Soil information transparency	SIT1	1.000				0.770	0.592	0.812	0.591	0.789
	SIT2	1.061	0.065	16.241	***	0.785	0.616			
	SIT3	1.033	0.064	16.130	***	0.751	0.564			
Water information transparency	WIT1	1.000				0.719	0.516	0.766	0.522	0.788
	WIT2	1.048	0.073	14.276	***	0.732	0.535			
	WIT3	0.938	0.068	13.794	***	0.717	0.514			
Competence trust	CT1	1.000				0.806	0.649	0.757	0.513	0.795
	CT2	1.178	0.085	13.882	***	0.705	0.497			
	CT3	0.995	0.081	17.019	***	0.627	0.393			
Benevolence trust	BT1	1.000				0.682	0.465	0.751	0.504	0.815
	BT2	0.889	0.103	8.643	***	0.632	0.399			
	BT3	1.185	0.123	9.691	***	0.797	0.635			
Online purchasing behavior	OPB1	1.000				0.789	0.622	0.791	0.557	0.765
	OPB2	0.876	0.058	15.232	***	0.722	0.521			
	OPB3	0.900	0.058	15.629	***	0.728	0.529			

***p < 0.001.

**Table 4:** Discriminant validity analysis.

	Soil information transparency	Water information transparency	Competence trust	Benevolence trust	Online purchasing behavior
Soil information transparency	0.769				
Water information transparency	0.656	0.722			
Competence trust	0.432	0.371	0.716		
Benevolence trust	0.405	0.284	0.301	0.711	
Online purchasing behavior	0.544	0.455	0.544	0.430	0.746

Note: Diagonals represent the square root of average variance extracted (AVE), and off-diagonals represent correlations.

## 4. Analysis of results and hypothesis testing

### 4.1. Structural equation modeling results

In order to continue to test the reliability of the hypotheses, this paper needs to test the degree of fit of the measurement model and the structural model based on the absolute and value-added fitness indicators of the model to ensure that the questionnaire data meet the expectations of the model as well as are identifiable. The results of the fit analysis of the measurement model and the structural model are shown in Table 5. The absolute and value-added fit indicators of the model are in the ideal and acceptable state, indicating that the model has a high fit.

**Table 5:** Results of the sample model fitness.

Fitness	Statistical test quantity	Ideal value	Model value	Evaluation
Absolute fit index	x2 /df	<3	1.6	Ideal
	GFI	>0.9	0.967	Ideal
	AGFI	>0.9	0.951	Ideal
	RMR	<0.05	0.051	Acceptable
	RMSEA	<0.08	0.034	Ideal
	NFI	>0.9	0.954	Ideal
Value-added fit index	RFI	>0.9	0.941	Ideal
	IFI	>0.9	0.982	Ideal
	CFI	>0.9	0.982	Ideal

**Figure 2:**
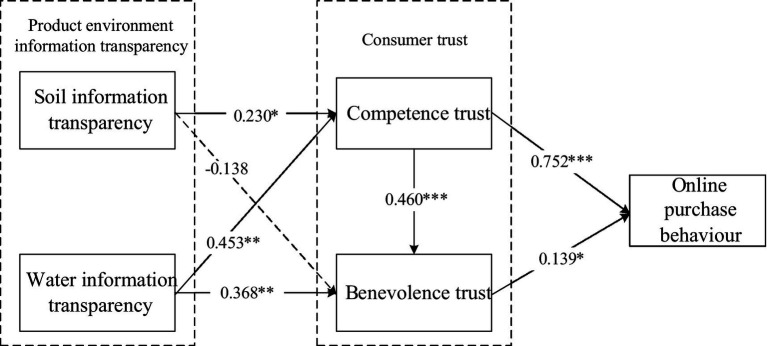
Model results. Dashed lines are non-significant, *p < 0.05, **p < 0.01, ***p < 0.001.

Amos 24.0 was used to test the relationship between soil information transparency, water information transparency, competence trust, benevolence trust, and online purchase behavior. The results of the path analysis are shown in Figure 2, hypothesis *H1a* is verified by the significant positive effect of soil information transparency on online consumer competence trust at the 5% level, indicating that for every 1% increase in soil information transparency, online consumer competence trust improves by 0.23%. Hypothesis *H2a* and *H2b* are both significantly positively correlated at the 1% level with coefficients of 0.453 and 0.368, respectively, and the hypotheses are supported. Hypothesis *H3a* and *H3b* are both significantly positively correlated at the 0.1% level with coefficients of 0.752 and 0.460, respectively, and both hypotheses are supported. Hypothesis *H4* is supported with a significantly positive coefficient at the 5% level. While *H1b* results show a negative correlation between soil information transparency and online consumer benevolence trust, indicating that the effect of soil information transparency on online consumers’ benevolence trust is inhibitive, which is inconsistent with the initial hypothesis and is rejected.

### 4.2. Discussion

#### 4.2.1. The effect of soil information transparency on online consumers’ competence trust and benevolence trust

According to the analysis of the model results in Figure 2, it is clear that soil information transparency has a significant positive effect on competence trust (*β* = 0.230, *p* < 0.05), and *H1a* is supported. This result is consistent with previous studies such as Chen (2007), Laureti and Benedetti (2018), and Mezger et al. (2020), in which consumers pay attention to the environmental and sustainability of the production process of agricultural products in terms of their impact on human health when making green produce purchases (Galati et al., 2022), the active disclosure of soil information transparency of green agricultural products by online sellers promotes consumers’ perception of the competence of that online seller’s operational skills and expertise. However, the effect of soil information transparency on benevolence trust is negatively insignificant (*β* = −0.138, *p* > 0.05), which is not consistent with the initial *H1b*. This result is similar to Fu et al. (2022), where information transparency of production process of green agricultural products could not significantly act on consumers’ benevolence trust. The possible reason is that consumers’ benevolence trust in sellers is mainly the result of sellers’ concern for consumers’ rights, which is difficult to promote through the product production process. Peschel and Aschemann-Witzel (2020) showed that a higher degree of transparency has little effect on product choice and even negatively affects it. Therefore, it is reasonable that soil information transparency does not significantly affect consumers’ benevolence trust in sellers.

#### 4.2.2. The effect of water information transparency on online consumers’ competence trust and benevolence trust

The model results show that water information transparency has a significant positive effect on online consumers’ competence trust (*β* = 0.453, *p* < 0.01), thus, *H2a* is supported. As Mullick and Das (2021) showed that irrigated Boro rice in Bangladesh has the greatest potential to meet sustainability needs and that people measure food security in terms of water resources (water quantity and quality) and believe that only an environment with few pollutants can have the capacity to provide high quality green agricultural products. Meanwhile, water information transparency also has a significant positive effect on online consumer benevolence trust (*β* = 0.368, *p* < 0.01), and *H2b* is supported. Sellers provide information on the quality of water in the production of green produce to ensure food safety and compliance with green product quality standards, thereby product environment transparency increases consumers’ trust in online sellers, in line with previous studies (Han and Yan, 2019; Castro-González et al., 2021; Giacomarra et al., 2021).

#### 4.2.3. The effect of online consumer trust on purchase behavior

The model results show that consumers’ competence trust significantly and positively affects online consumers’ purchase behavior (*β* = 0.752, *p* < 0.001) and benevolence trust also significantly and positively affects online consumers’ purchase behavior (*β* = 0.139, *p* < 0.05), and *H3a* and *H4* are supported, which indicates that online consumers’ trust can enhance their green agricultural products purchase behavior (Mohan et al., 2021; Fu et al., 2022). Interestingly, we found that competence trust has a stronger effect on online consumers’ purchasing behavior compared to benevolence trust. It may be that the higher the perceived competence trust of the online seller, i.e., the consumer believes that the seller has the appropriate competence, technology, and knowledge to produce green agricultural products that meet quality standards, and thus is willing to make a purchase behavior.

#### 4.2.4. The effect of competence trust on benevolence trust

The model results show that competence trust significantly and positively acts on benevolence trust, and *H3b* is supported (*β* = 0.460, *p* < 0.001). This indicates that the higher the consumers’ perception of competence trust of online sellers, then the higher the perception of benevolence trust. The pursuit of quality and production capacity of green produce by online sellers implies that they take into account the health and interests of consumers (Salvatore et al., 2022). Therefore, consumers’ trust in the competence of online sellers promotes benevolence trust (Fu et al., 2022).

## 5. Implications

### 5.1. Theoretical contributions

1. Based on the signaling theory, this study explores the influence of the signal of product environmental information transparency on the behavior of online green agricultural products consumers based on the characteristics of green agricultural products with high requirements for growing environment, which is an enrichment of the signaling theory. The results show that consumer trust in merchants is significantly enhanced by increasing the transparency of environmental information about green agricultural products. Thus, our study validates and extends the application of signaling theory in areas such as e-commerce market and green agricultural products in China.
2. Second, this study focuses on the effect of invisible product information disclosure. This complements previous research on the value of visible product information and provides new insights for achieving green agricultural products sustainability. In addition, this study enriches the research on product information transparency. Previous research on information transparency has mainly focused on supply chain transparency. This study aims to explore the information transparency of the environment in which green agricultural products are grown, thereby complementing existing research on product information transparency. Environmental information transparency is divided into soil information transparency and water information transparency, which is an extension of the environmental information transparency dimension. This extension helps to better understand which information disclosure of green agricultural products has a positive impact on consumer behavior. The results also show that different dimensions of environmental information transparency have different effects on different dimensions of online consumer trust, thus providing a theoretical basis for understanding the mechanisms that influence online green agricultural products purchasing behavior.

### 5.2. Managerial implications

1. Online sellers can increase consumers’ online trust through product information transparency. Product information transparency is proposed as a tool for producers to use in the online marketing of green agricultural products. Consumers’ access to information can be improved through online public disclosure of environmental quality indicators in the production process of green agricultural products. Transparency of product information can highlight the high safety and quality advantages of green agricultural products, reduce information asymmetry and thus promote consumer trust in sellers.
2. Consumers’ competence trust is a key factor influencing their online purchasing behavior of green agricultural products. Production operators can publicly share more invisible product information to demonstrate their strengths and increase consumers’ trust in the ability of online merchants, which in turn promotes online consumers’ green agricultural products purchasing behavior.
3. Benevolence trust also plays a very important role in online consumers’ purchasing behavior of green agricultural products. It is suggested that online merchants can increase consumers’ perception of benevolence trust by improving their service quality and disclosing production information, which in turn can effectively increase online consumers’ purchase of green agricultural products.

### 5.3. Policy implications

1. Government can encourage companies to disclose more information about the quality of agricultural products. Help enterprises to disclose information by subsidizing or opening free quality inspection departments. At the same time, the government can guide key leading enterprises to demonstrate product information disclosure and publicize the performance improvement after information disclosure through the media to motivate and encourage more enterprises to disclose invisible information.
2. For enterprises that grow green agricultural products on a large scale, the government can jointly build a green agricultural product traceability platform to achieve information transparency in the production process, safeguard the order of the green agricultural product market, and promote the sustainable development of the green agricultural product online consumption market.

## 6. Conclusion and limitations

### 6.1. Conclusion

The purpose of this study’s finding was to investigate the effect of product environmental information transparency on online consumers’ purchasing behavior of green agricultural products, which was chosen to be measured in two dimensions: soil information transparency and water information transparency. Soil information transparency has a significant positive effect on online consumers’ competence trust, but not on online consumers’ benevolence trust. Water information transparency has a significant positive effect on online consumers’ competence trust and benevolence trust. Both consumers’ competence trust as well as benevolence trust have a significant effect on online consumer purchase behavior. As Khan et al. (2022) showed that consumer trust is a key prerequisite for the establishment of a green product market, if product environmental information is communicated in a timely manner, it can increase online consumer trust and thus consumer purchase behavior.

### 6.2. Limitations and future research directions

Although the results of this study provide a reference for the sustainable development of green agricultural products, there are still some limitations. Firstly, this study used cross-sectional data, whereas longitudinal data can provide a more in-depth view of the relationship between product information transparency and consumer purchasing behavior, as consumer needs are constantly changing. Longitudinal research may be considered in the future, leading to new findings and conclusions. Second, this study selected environmental information transparency in green agricultural product information transparency, but product information that influences online consumer purchase behavior should be multidimensional. The influence of other dimensions of product information on consumer purchasing behavior, such as pesticide residues in the production process, hormone use and carbon emission indicators, can be explored in the future. In addition, as consumer heterogeneity may lead to differences in behavioral outcomes, the inclusion of other variables such as gender, education, age and other personal characteristics of consumers could be considered in the future to examine their control effects.

## Data availability statement

The raw data supporting the conclusions of this article will be made available by the authors, without undue reservation.

## Author contributions

SF: conceptualisation, investigation, conception, and design. RM: article revision and proofreading. GH: methodology and formal analysis. ZC: collected data and writing — original draft. HL: Writing — review and editing. All authors contributed to the article and approved the submitted version.

## Funding

This research was supported by the Social Science Foundation of Guangdong Province (#D22CGL18).

## Conflict of interest

ZC is employed by Chongqing Huadi Zihuan Technology Co.

The remaining authors declare that the research was conducted in the absence of any commercial or financial relationships that could be construed as a potential conflict of interest.

## Publisher’s note

All claims expressed in this article are solely those of the authors and do not necessarily represent those of their affiliated organizations, or those of the publisher, the editors and the reviewers. Any product that may be evaluated in this article, or claim that may be made by its manufacturer, is not guaranteed or endorsed by the publisher.

## Footnotes

^1^ https://www.tianqi.com/news/24610.html

^2^ http://www.199it.com/archives/1405773.html
